# Atmospheric Boundary Layer Wind Profile Estimation Using Neural Networks, Mesoscale Models, and LiDAR Measurements

**DOI:** 10.3390/s23073715

**Published:** 2023-04-03

**Authors:** Adrián García-Gutiérrez, Deibi López, Diego Domínguez, Jesús Gonzalo

**Affiliations:** Aerospace Engineering Area, Universidad de León, 24071 León, Spain

**Keywords:** machine learning, wind vertical profile, LiDAR, atmospheric boundary layer

## Abstract

This paper introduces a novel methodology that estimates the wind profile within the ABL by using a neural network along with predictions from a mesoscale model in conjunction with a single near-surface measurement. A major advantage of this solution compared to other solutions available in the literature is that it requires only near-surface measurements for prediction once the neural network has been trained. An additional advantage is the fact that it can be potentially used to explore the time evolution of the wind profile. Data collected by a LiDAR sensor located at the University of León (Spain) is used in the present research. The information obtained from the wind profile is valuable for multiple applications, such as preliminary calculations of the wind asset or CFD modeling.

## 1. Introduction

The atmospheric boundary layer is a challenging region to study in spite of the variety of existing models. The direct interaction of the ABL with the surface on time scales of less than an hour is a crucial factor in determining the wind speed profile within the ABL. The surface roughness, topography, and thermal properties all affect the ABL’s structure and dynamics, leading to a highly complex and variable wind speed profile. As a result, even small changes in local surface features can cause significant changes in the wind speed profile within the ABL. Nonetheless, the interest in studying and modeling it is considerable, since it plays a decisive role in multitude of different applications: wind resources, pollutant dispersion, air traffic, U-Space (European system to manage drone traffic), etc.

Among the various tools available for the study of ABL, wind sensors are indispensable [1,2]. Be it with anemometers, drones, balloons, satellites, or other instruments [3,4,5], a plethora of studies illustrate how such measurements are being used both by academia and industry. Vertical-profiling LiDAR [6] is of particular relevance as it provides the vertical wind profile with both high frequency and great accuracy. Nevertheless, they constitute an expensive instrument, which is why companies/research institutions cannot afford a large number of them.

Being in situ or remote sensing instruments, each one presents important gaps in range, resolution, refresh time, or cost. Therefore, the need for developing complete wind profiles from known partial information has been always relevant. Numerous models have arisen with different levels of complexity. Simple logarithmic laws based on terrain rugosity [7] and boundary layer development equations at global level [8] were first. The blooming of computers made possible the integration of full-physic equations of fluid motion at regional level [9]. These mesoscale models have been frequently used to study and analyze the ABL. One of the main limitations of mesoscale models is their temporal and spatial resolution, which may not be sufficient for many applications. The atmospheric boundary layer is highly dynamic and constantly changing, with turbulence and other small-scale processes occurring on very short timescales. Mesoscale models may not be able to capture these processes accurately, leading to errors in the predicted wind speed, temperature, and other meteorological variables. Another limitation of mesoscale models is their representation of surface features. The ABL is strongly dependent on local surface characteristics, such as topography, roughness, and land use, which can greatly influence the wind speed profile and other atmospheric variables. Modern computer fluid dynamic (CFD) techniques extend deeper into modeling terrain geometry, including obstacles, to provide unprecedented wind profile resolutions.

In micrometeorology, data assimilation is essential, but normally limited to surface information (stations on ground or buoys). Radiosondes, balloons, towers, radar and LiDAR instruments, airplanes, drones, and finally satellites are to some extent available to calibrate and improve models [10]. These processes are invaluable to keep accuracy at higher altitudes. 

In an attempt to reduce the economic cost, an earlier study by García-Gutiérrez [11] suggests the feasibility of monitoring multiple locations with a single LiDAR. To accomplish this, a neural network was used, being trained with the data collected by the LiDAR. In this way, it only was necessary to measure the wind near the ground to have the wind speed profile up to 300 m height.

Following that line of research, this study proposes to improve the accuracy of the ABL estimation algorithm employing mesoscale models, together with a new, improved, more efficient architecture for the machine learning (ML) algorithms.

The structure of this paper is as follows. In Section 2, experimental datasets together with machine learning numerical methods and the mesoscale model used in this study are presented. The methodology proposed is applied in Section 3, which presents and analyzes the findings of the study. Finally, in Section 4, the main findings are summarized together with their implications.

## 2. Materials and Methods

### 2.1. Wind Data

In order to train the numerical methods discussed in the subsequent sections, it is necessary to have a training dataset that is both sufficiently large and of high quality. To obtain these data, a vertical profiling LiDAR has been used, which is located in a suburban area of León (Spain). The period used for the training comprises between September 2021 and November 2022 (more than 47,000 samples). The LiDAR instrument used for the training is the ZephIR300 LiDAR (Campbell Scientific, Inc., Logan, UT, USA), validated in previous work [12], which can measure the wind speed/direction for a height between 10 and 300 m (32.8 and 300 ft). The ZephIR300 main features, according to the manufacturer, can be found in Table 1. In [12], the authors identified a mean bias of 0.1 m/s in the horizontal wind speed, while a mean bias of 2 degrees was identified in the wind direction.

This LiDAR uses continuous-wave technology, measuring 50 times a second at each altitude with constant sensitivity and averaging wind speed and direction values during periods over 10 min. This LiDAR uses continuous-wave technology, measuring 50 times per second at each altitude and averaging the wind speed and direction values during periods of 10 min. This type of continuous-wave system has less sensitivity at low signal-to-noise levels versus pulsed LiDAR.

In addition to the LiDAR, a ground station has been used to measure (at a height of 2 m) the following variables: wind speed (“U ground”), wind direction (“WD ground”), atmospheric pressure (“P”), ambient temperature (T), humidity, and rainfall. The weather station employed in this study is the AIRMAR WeatherStation 200WX (AIRMAR Technology Corporation, Milford, NH, USA), which utilizes ultrasonic technology to measure both wind speed and direction. Based on the manufacturer’s specifications, the accuracy of the wind speed measurements is ±5% at 10 m/s, while the accuracy of wind direction measurements is ±3° at the same speed. The AIRMAR WeatherStation 200WX is positioned in close proximity to the LiDAR, allowing for simultaneous measurement of meteorological variables at the same location. The roughness length for the measurement site has been estimated based on the Davenport roughness classification, which characterizes surface roughness according to the predominant type of terrain features present in the surrounding area. The estimated roughness length for the site is approximately 2 m, which is typical for regions with a mixture of low-rise and high-rise buildings. This suggests that the site is moderately rough, and the surrounding urban environment is likely to have a significant impact on the measured meteorological variables.

The measurements used in this study were subject to quality control (QC) procedures to ensure compliance with the International Electrotechnical Commission (IEC) standards and provide data of financial-grade quality. To achieve this, the ZephIR300 instrument applied a filtering process to remove data that did not meet the IEC compliance criteria. The filter code was used to indicate the reason for removing a particular data point.

### 2.2. Machine Learning Methods

One of the main objectives of this study was to determine which of the different machine learning methods is most suitable for the estimation of the atmospheric boundary layer. Many computational software packages dedicated to machine learning methods are currently available. As an example, one of the most widely used is scikit-learn [13]; version 0.24.2 was used for the current study. Some other alternatives include Keras [14] and TensorFlow [15].

Based on previous work in which machine learning methods have been used to estimate meteorological variables [16], several numerical models were selected for accuracy tests. These methods are:

- Simple multiple linear regressor [17]: a generalization of simple linear regression in cases where there is more than one independent variable.
- Ridge regressions [18]: this behaves like a simple linear regressor with an additional regulation method. The regulation term is to prevent overfitting, so the squares of the coefficients of the fitting method are considered in the loss function. Thus, high coefficients are penalized.
- Huber regressor [19]: a linear regression model that is more robust to outliers, owing to the use of a particular cost function.
- Decision tree regressor [20]: a method based on questions that narrows or restricts the range of possible values for the predictions by splitting the data into subsets.
- Random forest regressor [21] is an ensemble-based regression that trains many individual, uncorrelated decision trees with small depth. The assumption underpinning this technique is that several low-complex decision trees result in a more robust and consistent model by averaging all the output predictors of their individual trees.
- AdaBoost regressor [22] is a sequential machine learning technique used to randomly merge several weak learners from the dataset to produce a strong learner. Weak learners are trained by applying the particular machine learning algorithms. For each training dataset, a weight is attributed to each observation in the sample, and these weights are used to learn each hypothesis. False predictions are identified and assigned to the next learning base with a high weight on this incorrect prediction. The process loops until the algorithm is able to minimize the absolute value of the error. The median, or weighted mean, is used for the prediction of the individual base learner set.
- Gradient-boosting regressor [23]: gradient boosting is a more generalized version of the AdaBoost algorithm that enables the use of arbitrary cost functions, provided they are differentiable. Its flexibility has made it feasible to apply boosting to a multitude of problems (regression, multiple classification, etc.), making it one of the most widely used and successful machine learning methods. While there are several versions, the general underlying idea is similar: sequentially train models so that each model fits the residuals of the previous models.
- Bagging regressor [24]: in this algorithm, random sampling with replacement is used to train several models on random variations of the training set. The predictions of each model are averaged to obtain the final predictions.
- Multilayer perceptron: a neural network that has multiple layers. This method has been frequently used [25] for prediction of weather variables.
- Passive aggressive regressor [26]: passive aggressive algorithms are a family of machine learning algorithms that are popularly used in big data applications. Being an “online-learning” algorithm, the input data comes in sequential order and the machine learning model is updated sequentially.
- *k*-nearest neighbors (KNN) regressor [27]: a regressor that uses the average of the *k*-nearest neighbors of all features in the reference data set, weighted by their distance, for the prediction.

### 2.3. Mesoscale Model

In an effort to enhance the accuracy of the estimation, it is proposed to further optimize the ML algorithms by employing the mesoscale simulations results obtained using the Weather Research and Forecasting Model [28]. This numerical model has been widely used by industry and academia for weather forecasts, wind energy, and pollutant propagation applications [29,30,31,32], to name a few examples. The model executions are carried out in three nested domains with resolutions of 6 km (outermost domain), 3 km (middle domain), and 1.5 km (innermost domain), always fed by a global weather forecast such as the high-resolution Global Ensemble Forecast System (GEFS) [26]. The model configuration adopted for this study is supported by several years of experience in optimizing mesoscale models for wind engineering applications conducted by the research group [33,34]. The Mellor–Yamada–Nakanishi–Niino (MYNN) [35] level 2.5 scheme is used in combination with the MYNN surface layer, and the Noah land surface model is used as the planetary boundary layer. The Thompson–Graupel scheme of the WRF is chosen to account for microphysical processes and all runs are performed using the Dudhia scheme for the shortwave radiation scheme [36] with one-way mesh nudging for all domains. The model output is a 4D grid with a spatial coverage of 150 × 150 grid points at 1.5 km resolution, available in 38 equispaced vertical eta-levels, and time resolution of 1 h. The ensemble is composed of six members. For this study, the time series of the grid point closest to the respective location of interest, which was extracted from the innermost domain, was used. The simulations cover the same time period as the in situ measurements used in this study. They were launched every day at 3, 9, 15 and 21 h.

The computational cost of WRF simulation with three nested domains can vary depending on several factors, such as the size of the domains, the grid spacing, the physical parameterizations used, and the length of the simulation. Generally, it may require several hours to complete a 24 h simulation on a high-performance computing system with multiple processors. However, the exact computational cost can vary significantly depending on the specific configuration of the simulation and the available computing resources.

**Figure 1:**
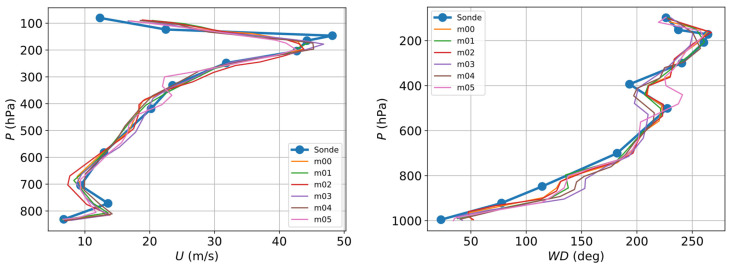
Comparison of the predictions by the mesoscale model and the weather balloons (sonde). The measurements were taken on 23 September 2019 at 00:00 UTC at the A Coruña airport.

To validate the mesoscale model, the predictions were compared with the results obtained by weather balloons. AEMET (State Meteorological Agency of Spain) weather balloons [37] are launched twice a day, every day, from the A Coruña airport at 00 and 12 UTC. The dataset consists of vertical profiles of temperature, dew-point temperature, wind speed, and wind direction from the surface to approximately 10^4^ Pa. As an example, the radiosonde data are compared against the mesoscale forecast prediction, as shown in Figure 1, where the prediction is an ensemble of several members with slightly different initial conditions before its propagation. These initial conditions are obtained from the ensemble GFS forecast products from the NOAA [38], so the model is able to capture the uncertainty associated with weather forecasting and provide a range of possible outcomes or scenarios.

In addition to the validation against the weather balloon, a cross-validation with the measurements obtained by the LiDAR and the METARs [39] at the airports listed in Table 2 was performed. These places are wide open areas without vegetation, specifically selected in the surroundings of airports in order to measure the wind profiles by the weather balloons. The most typical distribution of the ground is asphalt or very low vegetation. The results of the validation can be found in Table 3.

The validation campaign was conducted during a period of one month, from 23 September 2019 at 00:00 UTC to 23 October 2019 at 00:00 UTC. During this time, a total of 60 sounding balloons were launched (two per day). The METAR and LiDAR data were obtained at every hour, which were then compared to the output of the mesoscale models.

While sounding balloons can reach heights far beyond the ABL, their data were still included in the analysis because the ABL is heavily dependent on a multitude of factors such as wind shear and atmospheric waves, and thus an accurate representation of wind at all heights by the mesoscale model is required.

The results suggest that while the mesoscale models may perform well at higher altitudes, they are inadequate for accurately modeling the atmospheric boundary layer in regions with complex terrain. Thus, it is highlighted that more sophisticated models are needed that can account for the complex interactions between the ABL and the underlying terrain features.

## 3. Model Coupling

The operating principle of the different machine learning methods is essentially the same: based on a training dataset, the different models adjust the parameters in order to relate input variables to output variables.

**Figure 2:**
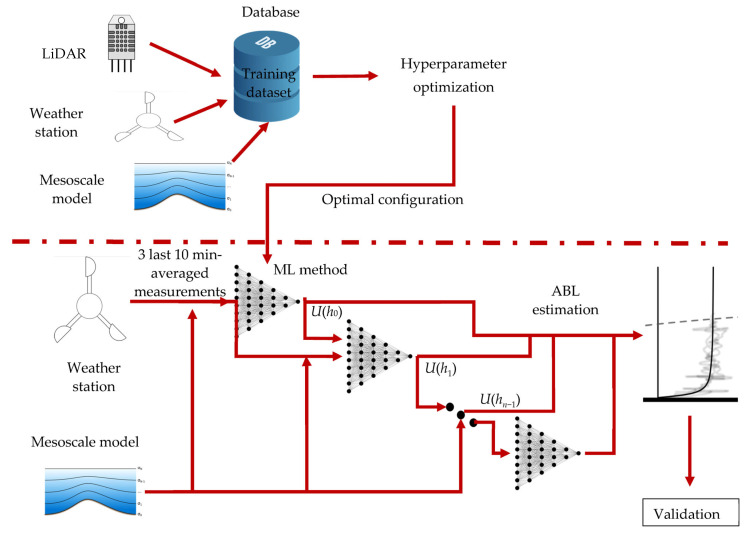
Dataflow for the proposed methodology.

This work (see Figure 2) presents two novelties with those found to-date for the ABL estimation using ML methods: (1) as input variables, not only the measurements taken by the weather station but also the predictions of the mesoscale model are included, which increases notably the amount of data to match; (2) to predict the velocity at height *i*, the prediction of the algorithm at height *i* − 1 is also considered.

Thus, once the LiDAR has collected a sufficient sample of data (it is shown later just what is sufficient), the proposed algorithm consists of:

1. Considering the weather variables measure by the ground station (the three last 10 min averaged measurements), together with the prediction of the mesoscale model (at *h*_0_), use the LiDAR data at height *h*_0_ to train the model (M0).
2. With that model, predict the value of the wind speed at *h*_0_. Train a new model to predict the wind speed at height *h*_1_ using the ground weather variables together with the prediction by the mesoscale model and the ML model M0.
3. Repeat for the *n*-levels.

Between step 1 and 2, a hyperparameter optimization must be performed to assure the optimality of the numerical methods used. Hyperparameters are parameters that are set before training a model, and they influence the model’s behavior and performance. The goal of this hyperparameter optimization is to find the best set of hyperparameters that produce the highest accuracy. This can be carried out through a combination of trial-and-error and algorithmic search methods such as grid search, random search, or Bayesian optimization. In this case, the grid search method was applied using Auto-Sklearn [40]. 

To determine the performance of the different methods, the following two metrics were used and are described below.

Mean absolute error (*MAE*) is defined as the mean of the absolute differences between the reference values and the predictions. It is given by the following equation:

$$MAE=\frac{1}{n}\sum \left|X_{i}-{\hat{X}}_{i}\right|,$$

in which $X_{i}$ is the measured data, ${\hat{X}}_{i}$ is the estimation, and n is the total number of measurements. Root mean square error (*RMSE*) is a metric used to determine how much a prediction deviates from the reference data. It gives more relevance to larger errors and has the following formula:

$$RMSE=\sqrt{\frac{1}{n}\sum {\left(X_{i}-{\hat{X}}_{i}\right)}^{2}}\;,$$

The Pearson coefficient is also considered by using the expression:

$$R=\frac{\sum \left(X_{i}-\bar{X}\;\right)\left(X_{i}-\bar{\hat{X}}\right)}{\sqrt{\sum {\left(X_{i}-\bar{X}\right)}^{2}\sum \left(\hat{X}-\bar{\hat{X}}\;\right)}}$$

in which $\bar{X}$ is the mean of the measured values and $\bar{\hat{X}}$ is the mean of the predicted values. In several applications, such as wind energy, it is not only relevant to reduce the error of the estimation in the individual values, but also to generate distributions of the values that are similar to those of the measured data.

A measure for the statistical similarity between two samples is the Kullback–Leibler divergence [41]. Given two probability density functions (*p*, *q*), it is calculated by:

$$D_{KL}\left(p,q\right)=\int_{\infty }^{\infty }p\left(x\right)log\;\left(\frac{p\left(x\right)}{q\left(x\right)}\right)\;dx.$$

The closer $D_{KL}$ is to 0, the more similar the two distribution functions. As this function is not symmetric, usually it is preferable to use the Jensen–Shannon divergence [42], given by:

$$JS\left(p,q\right)=0.5\;\left(D_{KL}\left(p,m\right)+D_{KL}\left(q,m\right)\right),$$

in which $m=\frac{1}{2}\left(p+q\right)$. Probability density function (PDF) estimation is important for several applications of interest, such as wind energy, risk management, climate studies, air pollution dispersion studies, etc. [43,44,45,46]. 

## 4. Results

The first step was to establish a set of standard conditions to compare the performance of each of the ML algorithms described above. For each algorithm, the average *MAE* error achieved using a two-month training dataset was used as the evaluation metric. Thus, for each combination of two consecutive months during the year, the method was trained with the measurements obtained in those months, and the *MAE* was calculated for the remaining months. Eventually, the average of all the values obtained was calculated.

A two-month period was chosen for the training set as a previous study [11] demonstrated a favorable balance between accuracy and economic feasibility, enabling up to six different locations to be monitored annually with the same LiDAR. Nevertheless, the trend observed with this training period was consistent with that of both shorter and longer training periods. The impact of the training set’s duration is examined in further detail later.

**Figure 3:**
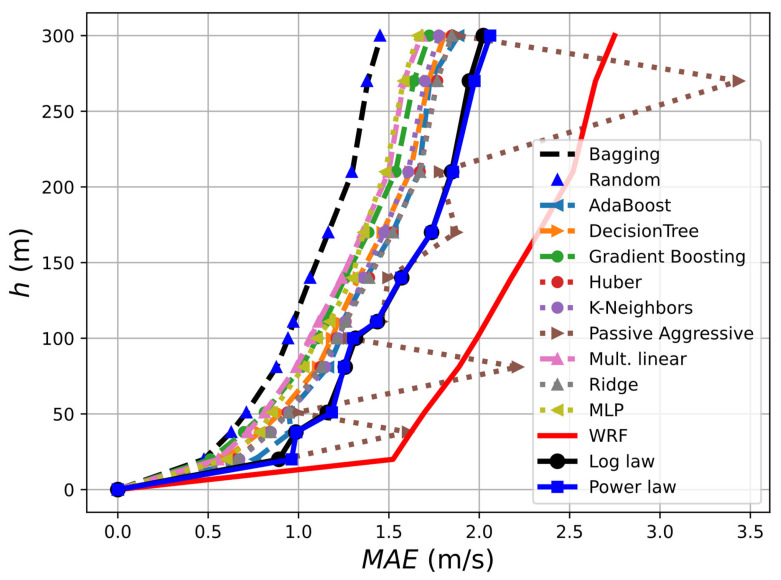
*MAE* error for different machine learning algorithms using 10 min averaged LiDAR measurements as reference and training data. The results obtained using only the mesoscale model are depicted by the “WRF” line.

Figure 3 shows the results obtained for the different methods. It follows that two methods stand out from the rest: bagging and the random forest regressors, which reveal superior behavior compared to the multilayer perceptron (MLP) regressor used in previous studies [11], and therefore proving to be better options for this application. Therefore, the results shown below in this section are obtained using only these two ML methods, since the other methods are found to be less accurate. They both obtain practically identical results, allowing a reduction in the error (compared to the other algorithms) by 15% for a height of 300 m, 19% for 150 m, and 14% at 50 m. The chaotic behavior of the passive aggressive algorithm also stands out, while the rest of the algorithms seem to have similar behavior, with the MLP as the third-most suitable. 

Additionally, in Figure 3, the performance of the mesoscale model (“WRF”) and two traditional methods of extrapolation of ABL are additionally included for comparison. The log law states that the wind speed (V) at a height (*z*) above the surface can be expressed as:

$$U\left(h\right)=U_{1}\;\frac{ln\left(\frac{h-d\;}{h_{0}}\right)}{ln\left(\frac{h_{1}-d\;}{h_{0}}\right)},\;$$

where *U*_1_ is the wind speed at a reference height ($h_{1}$), $z_{0}$ is the roughness length, and d is the zero-plane displacement. The power law, on the other hand, assumes that wind speeds increase with height according to a power law relationship. The power law states that the wind speed (*U*) at a height (*h*) above the surface can be expressed as:

$$U\left(h\right)=U_{1}{\left(\frac{z}{z_{1}}\right)}^{\alpha },$$

where α is the power law exponent. The parameters α, d, and $h_{0}$ were computed using least squares adjustment to the same dataset that were used to train the neural network.

**Figure 4:**
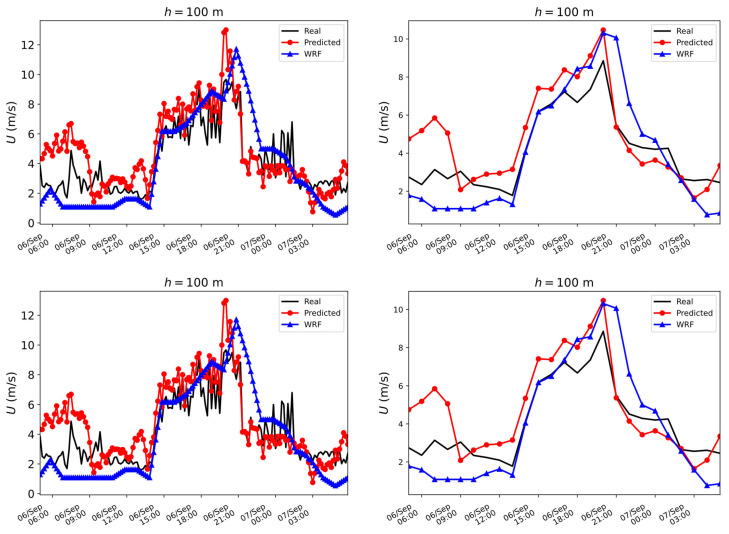
Simulated and real temporal series of the wind speed at h=81 m using the bagging regressor: (**Left**) 10 min averaged values; (**Right**) hourly averaged values.

In order to assist in the interpretation of what certain errors would imply, two time series of the *U* component during 6 and 7 September 2021 at a height of 81 m are plotted for illustrative purposes. The results can be seen in Figure 4.

**Figure 5:**
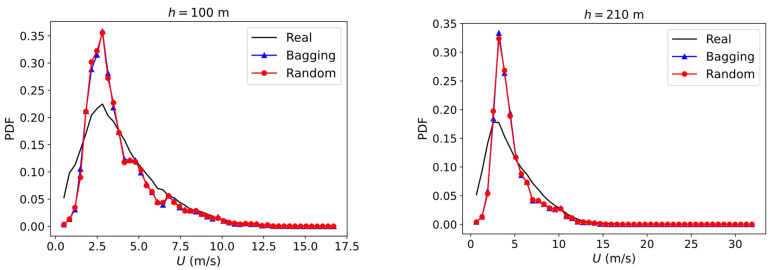
Comparison between measured and actual wind speed distribution.

Another issue that emerged was to determine how accurately the algorithm reflects the actual statistical distribution of the wind, as mentioned in the previous section. The comparison between the predicted and actual PDF can be found in Figure 5. Both the bagging and random forest regressor methods predict the distribution almost identically, showing that the most common real wind speed is also depicted by the numerical methods. In addition, another conclusion that can be drawn from the PDF figure is that these models have a worse prediction for lower wind speeds, tending to overestimate them and accumulating a greater frequency of the more common speeds. The statistic variables of the distribution are also shown in Table 4.

Then, once the ML algorithm that suits best for this application is chosen, the training data periods with consecutive months are studied as a function of the evolution of the errors at different heights during a whole year of predictions using the bagging regressor (Table 5). As expected, without exception, the errors are smaller closer to the ground and diminish the longer the training period. This error reduction is less pronounced for each month added to the training data. As an example, *MAE* decreases 21% at 100 m height from 1 to 2 months of training data but only 11% from 3 to 4 months; it is the same case for *RMSE*, which decreases 19% from 1 to 2 months but only 6% from 3 to 4 months at the same height. Similar results were obtained using the random forest regressor.

A comparison between the results achieved by the algorithm with and without the inclusion of the mesoscale model is of interest. Table 6 presents the results, indicating an improvement in mean absolute error (*MAE*) ranging between 6 and 11% for h = 100 m; 4 and 15% for h = 200 m; and 6 and 16% for h = 300 m. However, the percent of improvement is smaller for root mean squared error (*RMSE*), with oscillations between 3 and 5% for h = 100 m; 2 and 9% for h = 200 m; and 3 and 9% for h = 300 m.

**Figure 6:**
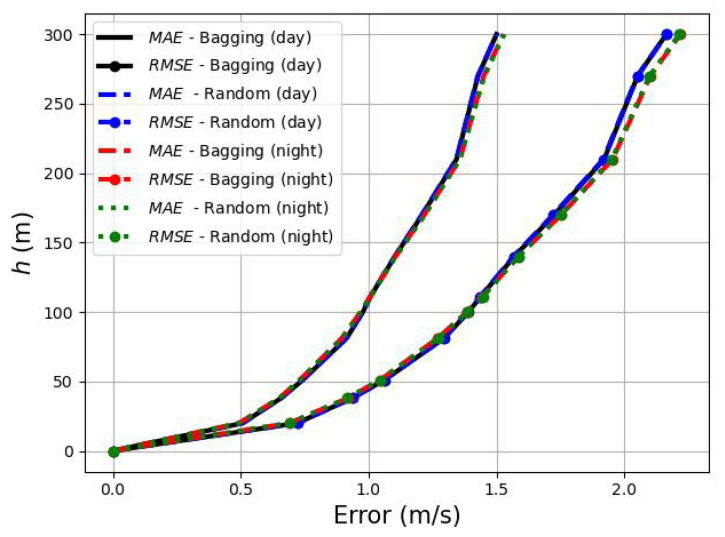
Averaged errors during day and night. The bagging and the random forest regressors obtained similar results.

In addition, all the data can be split during the day and night periods, and no remarkable differences are found between them. It can be seen in Figure 6, by means of the wind speed *MAE* and *RMSE* errors during the day and night, that both pairs of profiles are very close. At a height of 300 m, the maximum difference is observed, with the day *MAE* and *RMSE* errors being 2% and 1.8% lower than those at night.

**Figure 7:**
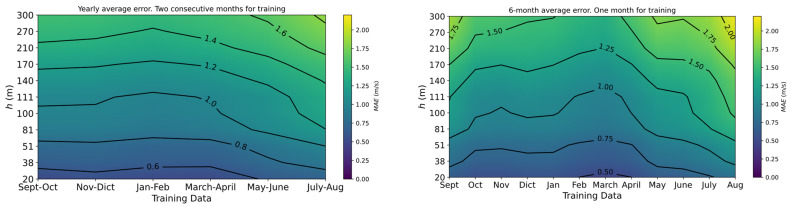
Seasonal effect on the wind speed *MAE*: (**Left**) regressor is trained with two consecutive months for a year of predictions; (**Right**) regressor is trained with one month for the next six months.

In addition, the effect of using different month configurations during the training period was also analyzed. As an example, a bagging regressor with two consecutive months for the training period and used for the whole season was taken as a reference for comparison in Figure 7 (left). Here, the figure shows the wind speed *MAE* over the year depending on the pair of months used for training. On the other hand (Figure 7 (right)), the regressor was trained with just 1 month data and is used during the next 6 months (simulating two different seasons during the year). 

This approach was carried out based on the factor of having the devices that gather the training data during the same amount of time during the year. As it can be observed comparing both graphs in Figure 7, the seasonal configuration has better performance during the end of winter and the beginning of spring, with lower *MAE* for the wind speed. Nevertheless, when the interest is mainly focused on mean accuracy during the whole year, the single regressor with consecutive training months is the best option.

## 5. Conclusions

Accurate atmospheric boundary layer characterization with sufficient spatial and temporal resolution is crucial for many applications. This study aimed to reduce the number of LiDAR instruments required for simultaneous monitoring of multiple locations. A novel methodology was developed to estimate the vertical wind profile of the ABL using a single point measurement near the ground and a mesoscale model to enhance accuracy. Machine learning algorithms were trained with real data from a LiDAR. This research was made possible by the ABL wind data collected by a LiDAR located at the University of León over the course of a year. 

Depending on the number of months (*n*) chosen for training, 12/*n* different locations could be monitored using just one LiDAR. Unlike previous studies, in this case, only one weather station on the ground surface is needed at each location to make the complete velocity profile prediction, once the regressor has been trained. 

After the regressors were fully defined and trained, they were able to estimate ABL wind profiles for heights up to 300 m above the ground. The results indicate that the bagging and random forest regressor methods were particularly effective, reducing errors by 15% to 19% when compared to other algorithms. This includes the multilayer perceptron used in previous studies [11]. Incorporating a mesoscale model has a positive impact on the algorithm’s performance, resulting in an improvement of up to 16% for specific heights and training datasets (Table 6). 

The accuracy of the algorithm was also determined by comparing the predicted and actual PDF. The bagging and random forest regressor methods predicted the distribution almost identically with an error in the mean of 1.7%. These data were also split into day and night periods and no significant differences were found. The effect of using different neural networks trained with different data due to seasonal effects was also analyzed. The results showed that the seasonal configuration had better performance in the end of winter and beginning of spring, with lower *MAE* in wind speed. However, for overall mean accuracy throughout the year, the single regressor with consecutive training months was the best option.

## Figures and Tables

**Table 1:** Main LiDAR characteristics according to the manufacturer. The accuracy is measured and calculated against a calibrated moving target.

Characteristic	Value
Range (m)	10–300
Height measurements (configurable by user)	10
Sampling rate (Hz)	50
Wind speed range (m/s)	1–70
Wind speed accuracy (m/s)	0.1
Wind direction accuracy (%)	<0.14%

**Table 2:** Airport locations.

Site	Name	GPS Location (deg)	Altitude (m)
1	A Coruña airport	42°53′47″ N 8°24′55″ W	370
2	Rozas airport	43°07′00″ N, 7°28′13″ W	440
3	Santiago airport	42°53′46″ N 8°24′54″ W	370
4	León airport	42°35′20″ N 5°39′20″ W	916

**Table 3:** Results of the mesoscale model validation.

Device	Location	Height	Variable	*RMSE*
Sounding balloon	A Coruña	1 km	Wind speed (m/s)	2.6
			Wind direction (deg)	7.7
			Temperature (K)	1.2
		3 km	Wind speed (m/s)	2.5
			Wind direction (deg)	8.5
			Temperature (K)	1.3
		5 km	Wind speed (m/s)	3.3
			Wind direction (deg)	8.2
			Temperature (K)	1.0
		7 km	Wind speed (m/s)	2.9
			Wind direction (deg)	6.7
			Temperature (K)	1.0
LiDAR	University of León	100 m	Wind speed (m/s)	3.1
			Wind direction (deg)	10
		200 m	Wind speed (m/s)	3.5
			Wind direction (deg)	11.5
		300 m	Wind speed (m/s)	4.0
			Wind direction (deg)	10.1
METAR	Airports (Table 2)	Surface	Pressure (kPa)	1.2
			Temperature (K)	2.1
			Wind speed (m/s)	1.7
			Wind direction (deg)	12.2

**Table 4:** Statistical variables for the distributions at h=210 m for both the bagging and random forest regressors.

	Real	Predicted	Difference (%)
Mean (m/s)	4.72	4.64	−1.7
Standard deviation (m/s)	2.8	2.24	−20.0
Skewness	1	1.46	+46.0
Kurtosis	4.07	5.1	+25.3

**Table 5:** Wind speed error variation as a function of the amount of training data for the bagging regressor.

Training Data:	1 Month			2 Months			3 Months			4 Months		
*h* (m)	100	200	300	100	200	300	100	200	300	100	200	300
***MAE* (m/s)**	1.11	1.66	1.89	0.88	1.30	1.45	0.81	1.20	1.32	0.73	1.07	1.17
***RMSE* (m/s)**	1.51	2.23	2.53	1.24	1.85	2.1	1.20	1.80	2.02	1.11	1.69	1.89
**R**	0.72	0.61	0.57	0.83	0.75	0.71	0.84	0.77	0.75	0.86	0.80	0.79
**JS**	0.26	0.18	0.20	0.16	0.14	0.14	0.13	0.05	0.04	0.10	0.03	0.03

**Table 6:** Quantification of the impact of the mesoscale model on the bagging regressor results.

		Bagging Regressor without Mesoscale Model				Improvements Achieved Using a Mesoscale Model			
	Training Data	1 Month	2 Months	3 Months	4 Months	1 Month	2 Months	3 Months	4 Months
*h* = 100 m	*MAE* (m/s)	1.19	0.94	0.89	0.82	7%	6%	9%	11%
	*RMSE* (m/s)	1.58	1.28	1.25	1.17	4%	3%	4%	5%
*h* = 200 m	*MAE* (m/s)	1.73	1.4	1.37	1.26	4%	7%	12%	15%
	*RMSE* (m/s)	2.28	1.95	1.94	1.85	2%	5%	7%	9%
*h* = 300 m	*MAE* (m/s)	2.00	1.56	1.53	1.4	6%	7%	14%	16%
	*RMSE* (m/s)	2.62	2.18	2.17	2.07	3%	4%	7%	9%

## Data Availability

Data can be accessed by contacting the corresponding author.
